# Supplementary material for: A pan-cancer analysis of homeobox family: expression characteristics and latent significance in prognosis and immune microenvironment
Source: Front Oncol. 2025 Feb 6;15:1521652. doi: 10.3389/fonc.2025.1521652 (PMC11840236; doi:10.3389/fonc.2025.1521652)
Supplement: Supplementary Figure 1 — Mutations of each HOX gene in 32 cancers (TCGA, PanCancer Atlas) by cBioportal. [file DataSheet1.zip › Suppl.files/raw code.zip!/code/4.survival/survivalplot.zip!/survivalplot/HOXC13_OS.SKCM.pdf]

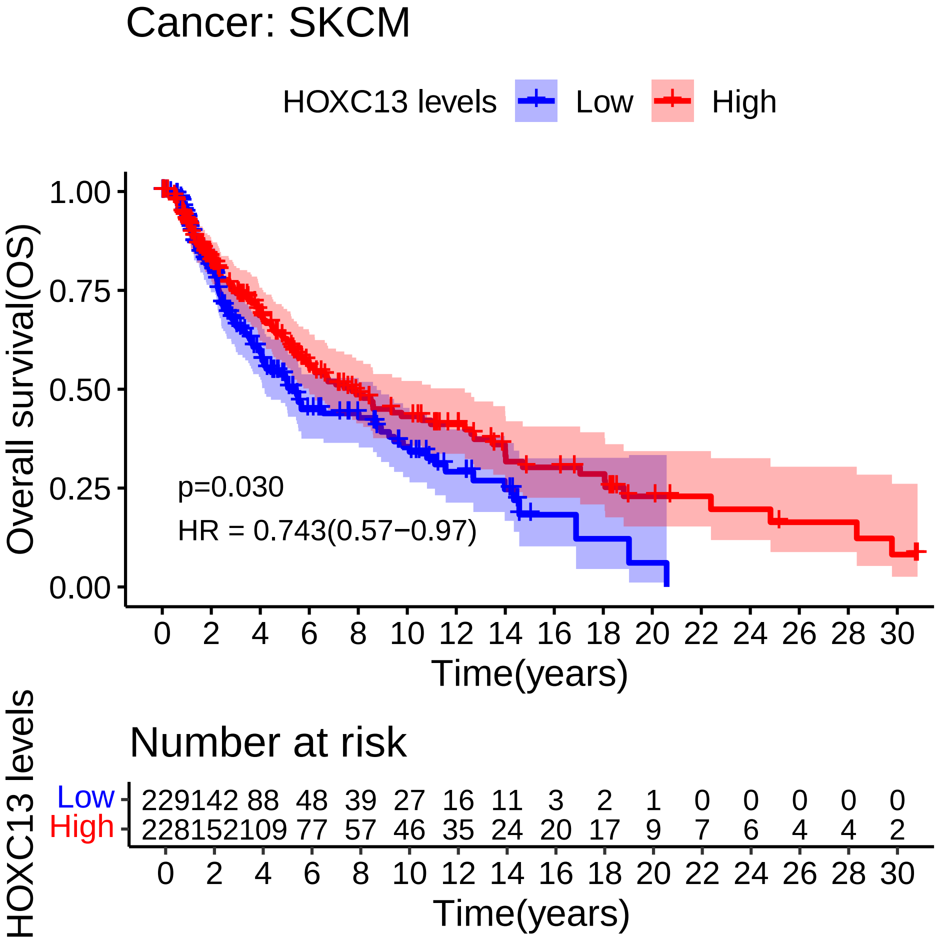

Cancer: SKCM
HOXC13 levels
Low
High
Overall survival(OS)
1.00
0.75
0.50
0.25
0.00
p=0.030
HR = 0.743(0.57−0.97)
0 2 4 6 8 10 12 14 16 18 20 22 24 26 28 30
Time(years)
Number at risk
HOXC13 levels
Low 229 142 88 48 39 27 16 11 3 2 1 0 0 0 0 0
High 228 152 109 77 57 46 35 24 20 17 9 7 6 4 4 2
0 2 4 6 8 10 12 14 16 18 20 22 24 26 28 30
Time(years)
